# The Impact of CSR Perceptions on Employees’ Turnover Intention during the COVID-19 Crisis in China

**DOI:** 10.3390/ijerph19148297

**Published:** 2022-07-07

**Authors:** Yang Cheng, Yuan Wang, Feihong Pan

**Affiliations:** 1 Department of Communication, North Carolina State University, Raleigh, NC 27695, USA; 2 Department of Media and Communication, City University of Hong Kong, Hong Kong, China; fhpan2-c@my.cityu.edu.hk

**Keywords:** CSR cynicism, China, COVID-19, turnover intention, distrust, expectancy violation

## Abstract

The COVID-19 pandemic has created tremendous challenges for organizations’ corporate social responsibility (CSR), communication, and relationship management with internal stakeholders such as employees. This study conducted an online survey of 466 employees working for large Chinese corporations during the pandemic. A structural equation model based on insights from expectancy violation theory was used to examine how negative violation valence increases employees’ turnover intention as mediated by uncertainty, CSR cynicism, and distrust. The survey results showed that employees’ negative violation valence positively influenced their uncertainty about their organizations’ CSR activities, which fostered their cynicism about CSR and distrust of their organization. Employees’ CSR cynicism increased their distrust toward their organizations, which increased their turnover intention. The theoretical and practical implications of the study are discussed as well.

## 1. Introduction

Recent research has shown that organizations and their management decisions can significantly affect stakeholders’ quality of life and physical and psychological well-being [1]. Stakeholders such as consumers, employees, and the community expect corporations to engage in corporate social responsibility (CSR) activities about environmental, social, and governance issues during crises [2]. For instance, the CVS drugstore gave up USD 2 billion in revenue when it stopped selling tobacco products to support public health [3]. Similarly, since the eruption of COVID-19 in China in December 2019, many corporations started active CSR activities. For instance, Microsoft in China donated over CNY 100 million to the Red Cross in Hunan Province, China on 26 January 2020 [4]. According to a survey conducted in China by McKinsey in March 2020, most of the participants and their corporations had participated in CSR activities, and around 39% of senior executives had deeply engaged in CSR activities during the pandemic [5]. By the end of January 2020, companies in China had donated a total of CNY 12.43 billion to support society during the pandemic [6]. Considering the critical role of large Chinese corporations’ CSR activities in fighting against the COVID-19 pandemic, it is meaningful to examine how their employees perceive their CSR efforts and its impacts.

Although many studies [7,8] have demonstrated the positive effects of CSR on firm performance and values, such effects could become contingent on stakeholders’ expectations. CSR activities during the COVID-19 pandemic, for example, might not satisfy individuals’ high expectations of large corporations (i.e., those with over 250 employees) [9] during crises if these corporations do not sacrifice short-term profits as part of their CSR efforts or these efforts do not directly benefit individuals [3]. Corporations’ CSR programs could face tremendous challenges if they fail to meet stakeholders’ expectations [2]. Scholars have established connections between stakeholders’ negative violation valence (i.e., the degree to which violations are considered to be negative expectations [10]), feelings of uncertainty, and attitudes toward corporate reputation [11]. Cheng et al. [12] found a negative relationship between consumers’ CSR skepticism and their brand loyalty and preferences. However, from an internal perspective, the relationships between employees’ negative violation valence, cynicism about corporate CSR, distrust, and turnover intention remain unclear. As the literature has shown, employees’ attitudes and behaviors significantly influence organizations’ crisis management, and more attention should be paid to internal employees during crises [13].

Scholars have found significant differences in corporations’ CSR activities in Western and Eastern contexts due to their different technological, cultural, and economic systems. For instance, Tang et al. [14] found that ethics were the most cited reason for CSR engagement on American corporations’ websites; employees and community were considered the most critical stakeholders in CSR communication. In Eastern contexts such as China, in contrast, CSR is regarded as ad hoc philanthropy, strategic philanthropy, or ethical business conduct, and employees are less emphasized [15]. Moreover, the Chinese public may have higher expectations of corporate CSR behaviors than American consumers [16]. Scholars [17,18,19] have found that in Asia, especially China, community and philanthropy are the main focus of corporate CSR activities. Shared values between the business and community are critically important in China for public perception. In contrast, in the U.S. CSR is a predictor of public trust in a business, and corporate social values might not affect stakeholders’ trust and supportive behavior. Consequently, CSR activities and stakeholders’ perceptions in an Eastern context such as China are distinct from those in the West, and corporate CSR communication and relationship management with internal stakeholders in the Chinese context deserve further exploration.

To fill the above research gap and enrich the crisis management literature, this paper draws on expectancy violation theory [20] and focuses on the global health crisis context. Due to the COVID-19 pandemic, employees’ work environment has been disrupted and turnover intention has become unpredictable [21]. The effectiveness of CSR communication within a crisis context in China has received limited attention thus far. This study aims to fill this gap by exploring the influence of CSR perceptions on employee relationship building and turnover intention in the context of the COVID-19 pandemic. The results have practical implications for CSR communicators and human resource managers during crises.

## 2. Literature Review

### 2.1. Theoretical Framework

#### 2.1.1. Expectancy Violation Theory

Expectancy violation theory (EVT) is a communication theory that explains how individuals deal with unexpected violations of social norms and expectations [22]. It was proposed by Burgoon and Jones [20], who used it to explain how individuals perceive and explain the invasion of their personal space. According to EVT, communication is an exchange of actions in which one person’s actions can violate the expectations of the other [20]. Participants in the exchange view this violation either positively or negatively, depending on their personal relationships or perceptions of the transgression [20]. This theory predicts that expectations will affect the outcome of communication interactions in a positive or negative manner, and the positive violations will strengthen the attractiveness of violators and vice versa [22].

According to Burgoon [23], expectations are consistent patterns of predictable behavior specific to individuals, contexts, and relationships. According to EVT, individuals use expectations to describe and frame their interactions with others, and these expectations dictate how they perceive such interactions and process information and influence their subsequent behavior [23]. When one behaves in a way that deviates from typical behavior, expectations are violated [24]. When an expectancy violation happens, an individual will pay more attention to the interaction process and deal with the violation through explanations and assessments [24].

EVT has previously been applied to the field of crisis communication. Scholars have used EVT to analyze business interactions between companies or between companies and consumers. Sohn and Lariscy [25] used EVT to examine how a CSR crisis affects a corporation’s reputation valence and found that higher expectations of the corporation due to its previous good reputation resulted in a more detailed investigation of expectancy violation behavior. Helm and Tolsdorf [26] documented that the public held higher expectations of companies with a better reputation and greater customer loyalty, and such companies tended to suffer more losses during crises than companies with a poorer reputation. Thus, when a company with a high reputation and customer loyalty violates customers’ expectations, customers may lose their trust in it.

According to Guerrero and Burgoon [27], if someone believes that violation valence and communicators’ reward valence are negative, he/she will reciprocate with negative behavior. Therefore, in the workplace, if an unpopular coworker is grouchy and unpleasant or if a disliked colleague is unhappy, people are more likely to be unhappy in return. In the business context, Kim [11] found that after an oil spill crisis at British Petroleum (BP), if BP’s stakeholders had a high prescriptive expectancy of environmental CSR activities, they had more negative reactions toward the firm due to negative violation valence. In this study, we hypothesize that if employees’ expectations of their organization’s CSR activities are negatively violated, they will become alert and evaluate the violator (i.e., organization) more negatively.

#### 2.1.2. Uncertainty

“Uncertainty” refers to a state of limited knowledge about existing states, future outcomes, or multiple possible outcomes [28]. Scholars have argued that violations have different effects on uncertainty, in that inconsistent negative violations increase uncertainty, whereas consistent violations (both positive and negative) lead to a decline in uncertainty [29]. As individuals’ expectations are based on organizations’ communications, media presence, and interactions with third parties, they expect certain kinds of behavior from an organization [30].

#### 2.1.3. CSR Cynicism

Davis [31] defined CSR as a company’s concern and responsibility for issues that involve duties beyond its legal, economic, and technical purposes. Story et al. [32] classified CSR as either internal or external. Internal CSR refers to the company’s responsibilities to internal stakeholders, such as improving working conditions [33]. External CSR is an organization’s responsibilities to stakeholders outside its organizational boundaries, such as the government, clients, local communities, business partners, and society [33]. Both internal and external CSR may induce cynicism from stakeholders [34]. CSR cynicism was conceptualized as “a stable individual characteristic of general disillusionment or apathy toward CSR across contexts” [35] (p. 219). According to Kuokkanen and Sun [36], even if external stakeholders have a positive attitude toward a hotel, its use of CSR for self-promotion can still induce skepticism from frequent travelers and elderly people. High levels of CSR cynicism occur when there is an incongruence between high external CSR and low internal CSR [37]. Cynicism occurs in both the internal and external business environment, and should be studied from a broader perspective covering all stakeholders.

#### 2.1.4. Employee Distrust

Trust is defined as “the willingness of a party to be vulnerable to the actions of another party based on the expectation that the other will perform a particular action important to the trustor” [38] (p. 712). Govier [39] defined distrust as the expectation that other individuals will not act in one’s best interests or will even engage in harmful behavior towards oneself. According to Cheng and Shen [40] (p. 2), stakeholders’ distrust toward an organization means “their fear of and perception of sinister intentions of the organization’s conduct related to the crisis”.

#### 2.1.5. Turnover Intention

Contrary to actual turnover, turnover intention refers to employees’ subjective attitudes about leaving the company or their perceived likelihood to change jobs in the near future [41]. Intention here means “a statement about a specific behavior of interests” [42] (p. 2760). Turnover intention has additionally been defined as “the extent to which an employee plans to leave the organization” [43] (p. 228), and serves as a major precursor of employee turnover. According to Ranjan and Yadav [44], employees’ turnover intention is affected by their cynicism, work engagement [45], work environment [46], quality of work experience [47], and organizational justice [48]. Researchers have studied turnover intention in various cultural contexts (e.g., individualistic and collectivistic cultures) and industries (e.g., hospitality and manufacturing) [49], as it is considered an effective warning indicator for problematic human resource management.

### 2.2. Development of Hypotheses

#### 2.2.1. Negative Violation Valence and CSR Uncertainty

The relationship between violation behavior and uncertainty has been explored by communication and management scholars. Smith [50] suggested that during a job hunt, a thank-you letter from the company could be seen as a positive violation of expectations as it can reduce uncertainty. Park et al. [51] documented that negative expectancy violation (e.g., a company’s violating customers’ higher CSR expectancy) leads to less positive attitudes and supportive behavioral intentions. According to Kim [11], a negative violation valence for an organization in crisis can increase uncertainty about its future performance. More specifically, people with more negative violation valence about BP had higher uncertainty about its future performance, placed more blame on BP for the oil spill, and perceived BP more negatively [11]. EVT indicates that negative violation leads to more negative evaluations of a violator and its future behavior [51]. Considering that CSR is an expectation that the public sets for large organizations [52], we argue that a large organization’s negative violation of its employees’ expectations about its CSR activities results in their uncertainty about these activities. Thus, we propose the following:

**Hypothesis** **(H1).** *Negative violation valence is positively related to uncertainty about CSR activities*.

#### 2.2.2. CSR Uncertainty and Cynicism

Scholars have examined the antecedents of employee cynicism. For example, Andersson [53] found that employee cynicism was influenced by employees’ self-esteem and locus of control. Employee perceptions of CSR may reduce their cynicism in the workplace [54]. In particular, employee skepticism about CSR is related to cynicism [55]. According to Pfrombeck et al. [56], social exchange theory can help explain organizational cynicism in a business setting. Social exchange includes employees’ uncertainty about the future actions of their organization, and may induce cynicism [56]. Therefore, if employees feel uncertain about their organization’s CSR activities, they are more likely to be cynical about these activities. Thus, we propose the following:

**Hypothesis** **(H2).** *Employees’ uncertainty about CSR activities is positively related to their CSR cynicism*.

#### 2.2.3. CSR Uncertainty and Distrust

Researchers have explored the relationship between CSR and trust/distrust. Lee [57] argued that internal CSR activities create a communal environment among employees, which improves their relationship with their organization, including trust. According to Lepoutre et al. [58], when there are uncertainties about CSR content and activities, employees become cynical about CSR. Employees’ feelings of uncertainty lead to indifference, which may affect their trust in the organization. Similarly, Jiang et al. [59] argued that when employees feel that CSR activities are hypocritical, this affects their trust in management. Thus, if employees feel uncertain about their organization’s CSR activities, they may distrust the organization. Therefore, we propose the following hypothesis:

**Hypothesis** **(H3).** *Employees’ uncertainty about their organization’s CSR activities is positively related to their distrust in the organization*.

#### 2.2.4. CSR Cynicism and Distrust

In addition to uncertainty, CSR cynicism may influence employee trust/distrust. Employees may have negative responses to CSR [35]. For example, employees may consider their organization’s CSR practice to be cynical and inauthentic [60]. Tourigny et al. [61] examined Chinese employees’ CSR perceptions and found that these perceptions affect their trust in their organization. According to Abraham [62], employees’ organizational cynicism leads to their mistrust. Similarly, we argue that employees who perceive their organization’s CSR efforts as cynical are more likely to distrust it. Thus, we propose the following hypothesis:

**Hypothesis** **(H4).** *Employees’ CSR cynicism is positively related to their distrust in their organization*.

#### 2.2.5. CSR Cynicism and Turnover Intention

Employees’ CSR perceptions may influence their turnover intention [63]. Positive internal CSR can help employees increase their employability [44], which may reduce their intention to leave their organization. Furthermore, Ranjan and Yadav [44] documented that employees are less willing to quit their organization if it has strong internal CSR performance, because an organization’s internal CSR reflects its welfare practices and procedural justice and therefore enhances its attractiveness, which negatively influences its employees’ turnover intention. Specifically, in a Chinese society, scholars found that CSR and ethical leadership demonstrate the value of organizations and further affect employees’ turnover intention [64]. In contrast, if employees perceive their organization’s lowly involvement with CSR activities or consider their firms’ CSR practice to be cynical, they may develop the idea of leaving their organization [64]. Therefore, we propose the following hypothesis:

**Hypothesis** **(H5).** *Employees’ CSR cynicism is positively related to their turnover intention*.

#### 2.2.6. Distrust and Turnover Intention

In addition to CSR cynicism, employees’ trust in their organization may be connected to turnover intention. Zeffane and Melhem [65] examined the factors that influenced employee turnover intention and found that employees’ trust had a negative effect on their turnover intention. Employees’ distrust triggers their negative feelings about their organization, which leads them to consider leaving as a way of decreasing these negative feelings [65]. Similarly, we argue that employees who distrust their organization may consider quitting. Thus, we propose the following hypothesis:

**Hypothesis** **(H6).** *Employees’ distrust in their organization is positively related to their turnover intention*.

## 3. Method

### 3.1. Data Collection

**Figure 1:**
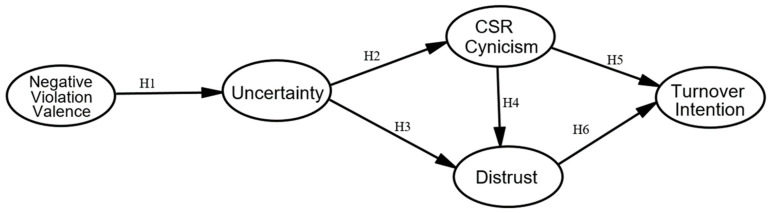
The conceptual model.

To test our hypotheses (see Figure 1), we conducted an online survey study. To enroll employees from large organizations (with more than 250 employees) [66] located in China, the researchers made use of the professional survey company Sojump, whose national panel contains 2 million members [67]. Upon approval from the ethics committee, we conducted a pretest in early February 2020 with 133 employees working in large organizations. The researchers then reviewed the dataset and calculated the reliability and validity of each measure. Cronbach’s α for the scale of each key variable ranged from 0.86 to 0.93. Consequently, we kept the original items and continued the process of data collection. At the beginning of the questionnaire, the respondents were provided with a detailed description of CSR activities. Several examples were presented, including offering supplies such as masks, paper towels, medicine, or food to those in need, providing cash donations to the people affected, and supporting employees affected by crises by providing short-term financial relief or paid vacations. In the survey section, attention-check items such as “if you are paying attention, kindly please choose ‘strongly agree’ to pass” were employed to ensure the quality of the online survey. From February to March 2020, 466 participants volunteered to join this study and completed the questionnaire. The data were collected for final analysis.

### 3.2. Participants

In the sample, the gender ratio was 51.5% female (*n* = 240) versus 48.5% male (*n* = 226). In terms of age, 81.5% (*n* = 380) of the respondents fell into the range from 25 to 39, 9.4% (*n* = 44) from 28 to 24, and 9% (*n* = 42) from 40 to 74. Among the 466 participants, 40.8% (*n* = 190) were in lower level management positions, followed by 26.6% (*n* = 124) in non-management positions, 29.6% (*n* = 138) in middle management positions, and 3% (*n* = 14) in top management positions. The average tenure of the respondents at their current company was approximately 4.3 years (*SD* = 3.5). Over one fifth of the participants (20.4%, *n* = 95) worked in manufacturing corporations, followed by information technology (19.7%, *n* = 92) and education companies (7.5%, *n* = 35).

### 3.3. Measures

All of the key variables were measured with a 5-point Likert scale ranging from “1” (strongly disagree) to “5” (strongly agree).

#### 3.3.1. Negative Violation Valence

This construct was gauged by adopting Kim’s [11] measures. Four items were used: “My organization’s CSR activities during the COVID-19 crisis makes me feel a lot worse about it”, “My organization’s CSR activities during the COVID-19 crisis make me feel it does not care about the society/community/environment”, “My organization’s CSR activities during the COVID-19 crisis makes me feel negatively about it”, and “My organization’s CSR activities during the COVID-19 crisis makes me feel very disappointed in it” (Cronbach’s α = 0.94).

#### 3.3.2. Uncertainty

To measure employees’ uncertainty about their organizations’ CSR activities, this paper adopted Kim’s [11] scale. Three items were included: “I feel a lot less confident about my organization’s CSR commitment”, “I feel a lot less confident about my organization’s social-friendly performance”, and “I become much less able to predict my organization’s commitment to society” (Cronbach’s α = 0.90).

#### 3.3.3. CSR Cynicism

Following the literature about CSR cynicism [68], we adopted three items to measure employees’ level of cynicism about their organization’s CSR activities: “I doubt that my organization’s CSR tries to solve social problems”, “I doubt that my organization’s CSR is actually socially responsible”, and “I doubt that my organization’s CSR follows the ethical responsibility to help society”. The level of reliability was 0.73.

#### 3.3.4. Distrust

To measure this construct, we applied the scale from Cheng and Chen [69] and utilized three items: “I feel that my organization will exploit my vulnerability given the chance”, “I feel that the way my organization is run is irresponsible and unreliable”, and “I feel that my organization cannot be trusted” (Cronbach’s α = 0.81).

#### 3.3.5. Turnover Intention

Following Lapointe et al. [70], we adopted three items to measure employees’ turnover intention: “I often think about leaving my organization”, “I am actively searching for a job at another organization”, and “If I could choose again, I would choose to work for another organization” (Cronbach’s α = 0.87).

#### 3.3.6. Control Variables

The literature has shown that the working experience of employees and perceived CSR fit influence participants’ CSR cynicism, distrust, and turnover intention [71,72,73]. Consequently, we controlled these variables in the final structural model. Work experience was measured by asking participants how many years they had worked in their organization. To measure CSR fit, we followed the scales of Moreno and Kang [71] and applied four items: “the CSR activities match our business goals”, “the CSR activities are relevant to the business goals”, “the CSR activities are a good fit for the business goals”, and “the CSR activities are compatible with the business goals”.

### 3.4. Statistical Procedures for Data Analysis

To test the six hypotheses, we performed correlation analysis and structural equation modeling (SEM).

## 4. Results

### 4.1. Descriptive Statistics and Correlation Analysis

To demonstrate the mean and standard deviation of each construct, we adopted the Likert scale, using “low” (1.00–1.99)”, “moderately low” (2.00–2.99), “neutral” (3), “moderately high” (3.01–3.99), and “high” (4.00–5.00) as the standard. The data indicated that respondents reported moderately low levels of negative violation valence (*M* = 2.60, *SD* = 1.26) and uncertainty about their organizations’ CSR activities (*M* = 2.82, *SD* = 1.24). In addition, employees reported a low level of agreement with distrust toward organizations (*M* = 1.99, *SD* = 0.88), CSR cynicism (*M* = 1.78, *SD* = 0.64), and turnover intention (*M* = 1.99, *SD* = 0.98). As shown in Table 1, these main constructs showed significant correlations to each other. The values of the inter-correlation coefficient between the constructs and the square root of average variance extracted (AVE) ranged from 0.08 to 0.88.

### 4.2. The Measurement Model

Before testing the hypotheses, we conducted a confirmatory factor analysis (CFA). The most conservative joint model testing criteria, according to Hu and Bentler [74], are as follows: the comparative fit index (CFI) is equal to or larger than 0.96 and the standardized root mean square residual (SRMR) is equal to or smaller than 0.10, the root mean square error of approximation (RMSEA) is equal to or smaller than 0.06, and SRMR is equal to or smaller than 0.10. The results of CFA analysis turned out to be satisfactory: *χ*^2^ = 134.55, *df* = 94, *p* < 0.001, *χ*^2^*/df* = 1.431, CFI = 0.992, TLI = 0.989, IFI = 0.992, SRMR = 0.031, RMSEA = 0.026 (90% CI = 0.018 − 0.042). The factor loadings of all items ranged from 0.64 to 0.91. According to Fornell and Larcker’s [75] criteria, if the value of composite reliability (CR) is 0.70 or larger and that of AVE is 0.50 or larger, the reliability of CR and AVE are considered to be acceptable. Our results suggested that the values of CR ranged from 0.73 to 0.94, and the scores of AVE ranged from 0.51 to 0.78, which indicated acceptable reliability. Furthermore, Fornell and Larcker’s [75] approach was employed to assess the discriminant validity of the measurement model. The square root of the AVE of each latent variable ranged from 0.71 to 0.88, higher than the correlations of any other latent variables (as shown in Table 1). Thus, discriminant validity was established and the data supported the reliability and validity of the applied measures.

### 4.3. Hypothesis Testing

**Figure 2:**
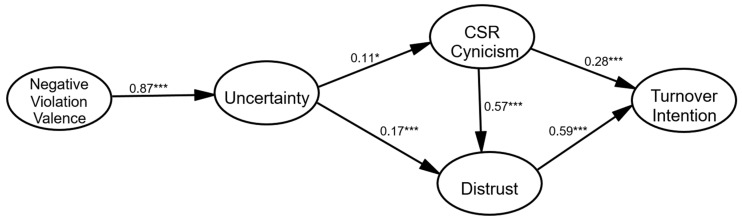
Results from the structural model. *Note.* Control variables in this model include the CSR fit and employees’ working experience. CSR refers to corporate social responsibility. *** *p* < 0.001. * *p* < 0.05.

To test the hypotheses, we employed a structural equation model for data analysis. The results indicated an excellent model fit: *χ*^2^ = 192.678, *df* = 123, *p* < 0.001, *χ*^2^*/df* = 1.566, CFI = 0.986, TLI = 0.980, IFI = 0.986, SRMR = 0.043, RMSEA = 0.035 (90% CI = 0.025 − 0.044). All of the path coefficients of the structural model are shown in Figure 2.

Specifically, H1 predicted a positive relationship between negative violation valence and uncertainty. The results showed that negative violation valence had a significant and positive impact on uncertainty about CSR (β = 0.87, *p* < 0.001), supporting H1. H2 further predicted that uncertainty exerts a positive impact on CSR cynicism, which was supported by the results (β = 0.11, *p* < 0.05). Uncertainty significantly positively influenced employees’ perceived distrust toward their organization (β = 0.17, *p* < 0.001), which means that a higher level of uncertainty about the organization’s CSR activity during the pandemic would lead to increased distrust from internal employees. Thus, H3 was supported. Hypothesis 4 posited that CSR cynicism is positively related to distrust. The data demonstrated that a significant and positive impact flow existed between CSR cynicism and distrust (β = 0.57, *p* < 0.001), supporting H4. In addition, the results showed that CSR cynicism significantly positively affects turnover intention (β = 0.28, *p* < 0.001). Thus, H5 was supported. Finally, employees’ distrust led to their intention to quit their job (β = 0.59, *p* < 0.001), supporting H6.

Indirect effects in the current model were estimated through a bootstrapping procedure (*N* = 5,000 samples) via Amos 20. Negative violation valence had a significant and positive indirect effect on CSR cynicism through the mediation of uncertainty (β = 0.11, *p* = 0.040, BC 95% CI: 0.01 to 0.21). Uncertainty significantly mediated the relationship between negative violation valence and distrust (β = 0.23, *p* < 0.001, BC 95% CI: 0.13 to 0.32). Negative violation valence had a positive indirect effect on turnover intention through the serial mediation of uncertainty, CSR cynicism, and distrust (β = 0.17, *p* = 0.001, BC 95% CI: 0.09 to 0.25). Moreover, uncertainty had a positive indirect effect on turnover intention through the channel of distrust and CSR cynicism (β = 0.19, *p* = 0.001, BC 95% CI: 0.10 to 0.28).

## 5. Discussion

The prolonged COVID-19 pandemic has created tremendous challenges for organizations’ CSR practices and their relationships with employees. This study surveyed 466 employees working in large organizations in China. It found that employees’ negative violation valence increased their uncertainty about CSR activities, which fostered their cynicism about CSR and distrust in their organization. Employees’ CSR cynicism strengthened their distrust toward their organization, which increased their turnover intention. In this section, we discuss the theoretical and practical implications of these findings.

First, the results of this study indicate that expectancy violation theory is a useful theoretical framework for explaining employees’ CSR attitudes toward organizations during a crisis. This theory provides a new understanding of the effectiveness of CSR communication in a pandemic. One of the major findings of this study is that employees’ negative violation valence increases their uncertainty about their organization’s CSR activities, which is consistent with previous research [11]. If employees are unsatisfied with their organization’s CSR activities during the COVID-19 pandemic, they are more likely to feel uncertain about the organization’s commitment to CSR and society. Thus, large organizations should make more efforts to improve their CSR activities and satisfy employee expectations during a public health crisis so that they can retain their employees’ confidence in their commitment to CSR and society. Although scholars have investigated the general public’s and consumers’ perceptions of organizational CSR practices [11,51], they have seldom examined the perceptions of internal stakeholders such as employees. Thus, our findings fill this gap by focusing on employees’ perceptions of organizational CSR practices and their consequences.

Second, as trust is considered to be a dimension of employee–organization relationships [76], this study is one of the few studies to integrate the CSR perspective with the relational perspective by documenting the effects of CSR uncertainty and cynicism on distrust. It thus advances the literature on CSR and employee–organization relationships. One important finding is that employees’ uncertainty about their organization’s CSR activities results in CSR cynicism and distrust in their organization. If employees feel less confident about their organization’s CSR commitment, they are more likely to doubt that their organization’s CSR is actually socially responsible or addresses social problems, and will consequently develop distrust in their organization [77]. Furthermore, this study found that employees’ CSR cynicism positively influenced their distrust in their organization. As previous literature has indicated, CSR activities reflect company ethics and values and help organizations to act as “trustees” for the interests of employees [78] (p. 10). With the implementation of CSR activities, employees can fulfill their own social needs and become enthusiastic toward their work, as CSR helps to reach goal congruence and a common identity between employees and their organization [64]. Consequently, if employees develop their own cynicism toward their organizations’ CSR, the most proximate outcome would be distrustful perceptions. Thus, large organizations should therefore promote their CSR activities to their employees and demonstrate their CSR commitment, which can induce their employees to trust them.

Third, the findings of this study contribute to the business literature by shedding light on the influence of public relations practices on organizations. The results demonstrate that employees’ cynicism about their organization’s CSR activities and distrust in the organization increases their turnover intention; these findings are similar to those in previous research [44]. If employees doubt that their organization’s CSR helps society or solves social problems, they are more likely to consider leaving the organization. Therefore, this study advances the CSR literature by identifying employee CSR uncertainty as a new factor that may influence turnover intention. Moreover, employees who distrust their organization are more likely to leave it to work for another organization. This finding is critical, especially during the COVID-19 pandemic, because this global crisis has brought challenges to organizational management in terms of maintaining employees’ trust in their organization [79]. Thus, large organizations could strengthen their CSR efforts (e.g., delegating a specific division for CSR practices, offering paid hours for employees’ CSR engagement, or enhancing CSR fit with their strategic goals) to maintain their employees’ trust, which can help with employee retention. 

Practically, the results of this study provide important managerial implications for large organizations. The proposed model can be used to measure the effectiveness of organizational CSR practice. The COVID-19 pandemic has significantly impacted stakeholders in organizations, such as employees. Managers can apply this tool to focus on employees’ perceptions of CSR communication in order to examine their expectations concerning current CSR activities and evaluate whether their activities will have a negative or positive valence. The model suggests that if CSR activities do not satisfy employees’ expectations during a pandemic, then CSR cynicism and distrust toward organizations could occur, leading to increased turnover. Consequently, examining and monitoring employees’ perceptions of CSR activities is beneficial for human resource management and internal crisis communication. Large companies might develop adequate measures or structures to ensure that their employees can help the companies to perform CSR work in a positive and mentally comfortable manner rather than forcing them to complete CSR activities. Leaders in firms should listen to employees and carry their interests in mind when they encourage and conduct CSR activities. An ethical and healthy leadership style and “goal congruence” between a firm and its employees can reduce employees’ turnover intention [64].

In addition, this study shows that positive relationship management with internal employees might reduce employee turnover costs. During a crisis, relationships between organizations and employees are threatened [80]. If corporations ensure that their CSR activities are perceived as beneficial, ethical, and supportive during a crisis, employees will be confident about such activities and trust their organization, which will significantly reduce turnover intention.

### Limitations and Directions for Future Research

Although this study contributes to the literature on CSR and internal employee management during the COVID-19 pandemic, several limitations must be stated here. First, this study only focused on internal communication within big Chinese organizations. Future studies could employ comparative methods to further validate the proposed hypotheses and explore cultural or contextual factors that might influence employees’ perceptions of CSR during crises and the impact on turnover intention. For example, they could examine whether the types of companies (i.e., state-owned enterprises and private companies) might influence the relationship between employees’ CSR perceptions and their distrust and turnover intention. Second, as the magnitude of violation valence might influence perceived uncertainty, future scholarship could continue to explore how employees’ reactions to a violator, such as the organization during a crisis, might influence their trust in organizations and work performance. Third, employees’ individual differences might influence their perceptions of an organization during a crisis. Future work could investigate how CSR involvement or value orientations [81] predict distrust and CSR cynicism.

## 6. Conclusions

This paper presented a timely study on CSR perceptions, distrust, and turnover intention of employees in crises of contemporary Chinese society. It contributed to the body of knowledge on CSR in building trust and shed light on the application of ethical CSR in crisis communication of China. This study also built on the most up-to-date literature and addressed an important issue on CSR practice in China from an internal employee’s perspective.

## Figures and Tables

**Table 1:** Inter-correlation between the Constructs and the Square Root of AVEs (Fornell–Larcker Criterion).

Constructs	Negative Violence Valence	Uncertainty	CSR Cynicism	Distrust	Turnover Intention
Negative Violence Valence	**0.88**				
Uncertainty	0.80 **	**0.87**			
CSR Cynicism	0.11 **	0.08	**0.71**		
Distrust	0.27 **	0.18 **	0.45 **	**0.76**	
Turnover Intention	0.25 **	0.24 **	0.49 **	0.64 **	**0.83**

*Note.* ** Correlation is significant at the 0.01 level (2-tailed). CSR refers to corporate social responsibility; AVE refers to average variance extracted. Values in the diagonal bolded are the square root of AVE while the off-diagonals are correlations.

## Data Availability

The data are not publicly available due to privacy or ethical restrictions.
